# Cancer and Nutrients

**DOI:** 10.3390/cancers15164151

**Published:** 2023-08-17

**Authors:** Hardeep Singh Tuli, Mukerrem Betul Yerer, Vikas Yadav

**Affiliations:** 1 Department of Bio-Sciences and Technology, Maharishi Markandeshwar Engineering College, Maharishi Markandeshwar (Deemed to be University), Mullana-Ambala 133207, India; 2 Department of Pharmacology, Faculty of Pharmacy, Erciyes University, Kayseri 38039, Turkey; eczbetul@yahoo.com; 3 Department of Translational Medicine, Clinical Research Centre, Skåne University Hospital, Lund University, SE-20213 Malmö, Sweden; vikasyadav40@gmail.com

Editorial: Over the last few decades, the scientific community has recognized the enormous potential of bioactive dietary nutrients/components in the management and prevention of cancer. Based on the fact that bioactive dietary nutrients and molecules modulate the key deregulated signaling pathways in cancers, including proliferation, apoptosis, cell cycle progression, migration, invasion, and angiogenesis [1,2,3], it is hypothesized that 30–70% of all cancer cases could be prevented. These dietary components also have anti-inflammatory properties linked to their inhibition of cytokines or inflammatory mediators [4,5]. Understanding how these molecules interact with their respective oncogenic targets and the underlying molecular mechanism(s) is crucial for developing an efficient cancer therapy strategy based on dietary chemicals. The scientific community will be inspired to develop novel anti-tumor medications with cutting-edge formulations for clinical testing by the research findings from such discoveries. The goal of this Special Issue was to encourage researchers to submit original research and review articles on the subject of medically significant bioactive nutrient molecules and their ability to prevent tumors. To reduce the risk of colorectal cancer, Seyyedsalehi et al. investigated dietary changes that would enhance sources of betaine and manage the usage of animal products as sources of sphingomyelin or other types of choline. On average, social dining should be avoided until 12 months after head and neck cancer therapy, according to Ninfa et al. Another study by Dhillon et al. revealed that measuring the concentration profiles of specific micronutrients may help identify men who are at a high risk of developing prostate cancer. Additionally, this approach may assist in designing future dietary intervention trials to lower the risk of developing this deadly disease. Similarly, Sun et al. investigated the relationship between nutrient intakes associated with lung cancer among underrepresented study populations and enhanced the risk assessment of lung cancer. A systematic evaluation of dietary polyphenol intake by Fagundes et al. revealed a decrease in the risk of gastric cancer, with females experiencing the highest decline. Further research examining polyphenol consumption and gastric cancer in populations in Latin America is also necessary. Interestingly, Casirati and colleagues have reviewed the concept of “immunonutrition”, which encompasses the utilization of specific nutrients to enhance the immune system and enhance postoperative results, particularly after bladder surgery. This Special Issue also includes a study of luteolin’s synergistic method of action, insights into nanodelivery, and all conceivable cellular interactions in cancer. We sincerely hope you enjoy reading the articles on this research topic, which address a subject that will likely become even more decisive in the coming years.
